# Transcriptome analysis confers a complex disease resistance network in wild rice *Oryza meyeriana* against *Xanthomonas oryzae* pv*. oryzae*

**DOI:** 10.1038/srep38215

**Published:** 2016-12-01

**Authors:** Xiao-Jie Cheng, Bin He, Lin Chen, Su-qin Xiao, Jian Fu, Yue Chen, Teng-qiong Yu, Zai-Quan Cheng, Hong Feng

**Affiliations:** 1 Key Laboratory of Bio-resources and Eco-environment, Ministry of Education, Sichuan Key laboratory of Molecular Biology and Biotechnology, College of Life Sciences, Sichuan University, Chengdu 610064, Sichuan, P. R. China; 2 Biotechnology & Genetic Resources Institute, Yunnan Academy of Agricultural Sciences, Kunming 650223, Yunnan, China

## Abstract

Rice bacterial blight (BB), caused by *Xanthomonas oryzae* pv. *oryzae (Xoo*), is one of the devastating diseases of rice. It is well established that the wild rice *Oryza meyeriana* is immune to BB. In this study, the transcriptomic analysis was carried out by RNA sequencing of *O. meyeriana* leaves, inoculated with *Xoo* to understand the transcriptional responses and interaction between the host and pathogen. Totally, 57,313 unitranscripts were *de novo* assembled from 58.7 Gb clean reads and 14,143 unitranscripts were identified after *Xoo* inoculation. The significant metabolic pathways related to the disease resistance enriched by KEGG, were revealed to plant-pathogen interaction, phytohormone signaling, ubiquitin mediated proteolysis, and phenylpropanoid biosynthesis. Further, many disease resistance genes were also identified to be differentially expressed in response to *Xoo* infection. Conclusively, the present study indicated that the induced innate immunity comprise the basal defence frontier of *O. meyeriana* against *Xoo* infection. And then, the resistance genes are activated. Simultaneously, the other signaling transduction pathways like phytohormones and ubiquitin mediated proteolysis may contribute to the disease defence through modulation of the disease-related genes or pathways. This could be an useful information for further investigating the molecular mechanism associated with disease resistance in *O. meyeriana*.

Rice (*Oryza sativa* L.) is one of the most important food crops in the world. However, rice production is seriously constrained by various diseases^1^. More than 70 diseases caused by fungi, bacteria, viruses, and nematodes have been reported on rice^2^. Bacterial leaf blight (BB) caused by *Xanthomonas oryzae pv. oryzae (Xoo)* has been become a devastating disease of rice worldwide in 1960s and 1970s when the high-yielding varieties were introduced^3^. In practice, development of the resistant cultivars and transgenic rice is one of the major strategies for BB management^1^,^2^. Nevertheless, the high degree of pathogenic variation in *Xoo*^4^ and rapid loss of resistance in the rice varieties against BB^5^, development of resistant varieties become a new challenge for breeders. Therefore, exploring new resistant genes become an urgent and arduous task both in practice and theory.

Wild rice, which is growing widely in 77 countries of the Asia, Africa, Latin America and Australia, is considered to be an important and rich gene pool^6^. Many species of wild rice may possess substantial elite genes that are resistant to the diseases and insects^6^,^7^. Plant breeders and geneticists of the world have made great progress to identify the resistant materials to the rice BB. Until today, more than 40 *Xanthomonas* resistance (*Xa*) genes specifically resistant to various strains of *Xoo* were identified or isolated from a variety of rice materials^8^. Some of these genes were obtained from different wild rice species. For example, *Xa21* is the first resistant gene derived from the wild rice (*O. longistaminata*)^7^.

The awnless wild rice species of *O. meyeriana* Baill is a unique source^9^ (Fig. 1a), possessing GG genome that is different from the cultivated rice with AA genome^10^. *O. meyeriana* collected from different regions of South China has been demonstrated highly resistant to *Xoo* infection, usually accompanied by the typical hypersensitive response (HR)^11^. In our study, disease spot was restricted to the inoculated points on the leaves after inoculated with *Xoo* (Fig. 1a). In contrast, the susceptible cultivar of *O. sativa* showed spreading lesions after inoculation with the same bacterial pathogen (Fig. 1a). Previous studies on the physiological and anatomical changes of *O. meyeriana* after *Xoo* inoculation revealed that xylem cell wall was thickened and nitric oxide (NO) level was raised after *Xoo* infection^12^,^13^. Therefore, *O. meyeriana* is regarded as a valuable gene pool. The genetic resources of *O. meyeriana* have been successfully used for BB resistance through asymmetric somatic hybridization^14^. As a result, highly resistant lines were selected from the hybrid progeny between *O. meyeriana* and *O. sativa* subsp. japonica^15^. In addition, several molecular approaches were also used to identify the resistance-related genes to BB. For example, some genes involved in cell wall lignification were identified in *O. meyeriana* in response to the *Xoo* infection by the suppression subtractive hybridization^16^. Most recently, a transcriptomic sequencing also illustrated that many putative resistance-related genes were transcribed in *O. meyeriana* under the normal growing condition^17^. Although these studies provide valuable information with regards to the BB resistance, available information concerning molecular mechanism or genes involved in the response to *Xoo* infection for *O. meyeriana* is still limited.

It is well documented that plants have evolved complex innate immune systems in response to the biotic and abiotic stresses. The plant immune system is composed of surveillance systems to perceive the microbe elicitors, such as the well-characterized flagellin and peptidoglycan, which in turn allow plants to switch from the normal growth and development to a defence mode^18^,^19^. This process is so called as pathogen-associated molecular patterns (PAMP)-triggered immunity (PTI), which is considered as an important initial level in pathogen response. However, the bacterial pathogens may inhibit PTI via secreting virulence factors (effectors) into the plant cells^19^. To overcome these virulence factors, plants have evolved disease resistance (*R*) genes, typically encoding the nucleotide-binding site and leucine-rich repeat (NBS-LRR) domains, to recognize these effectors and initiate a second layer of response called the effector-triggered immunity (ETI). ETI often leads to HR mediated by the *R* proteins in plant and is an accelerated and amplified PTI response, resulting in disease resistance to protect the plant itself^19^. However, non-host resistance to pathogens is often associated with the HR, in which the molecular recognition events and activation of plant innate immunity are involved^20^. Besides, the other signaling pathways like phytohormone signaling, ubiquitin mediated proteolysis, etc, are involved in the defence response to the pathogens in plants, too^21^. Overall, plant defence response is a complex process, involving multiple signaling pathways and their cross-talks.

Transcriptomic sequencing based on the next-generation sequencing technologies, provides a powerful and effective strategy to produce genome-scale sequence data, which allows the whole transcriptome to be investigated in a high-throughput and quantitative manner, especially for non-model organisms with genomic sequences that are yet to be measured^22^. Therefore, RNA-seq has been successfully used to investigate the differential gene expression in many pathosystems, like *Xoo* in cultivar rice^23^, *X. axonopodis* pv. *glycines* in soybean^24^, or *X. arboricola* pv. *pruni* in peach^25^. To get insights on the global response of *O. meyeriana* to *Xoo* infection, deep RNA sequencing was performed in this work. Through identifying differentially expressed genes (DEGs) of *O. meyeriana* after *Xoo* infection, the major metabolic pathways related to disease resistance including the plant innate immunity, phytohormone signaling, ubiquitin mediated proteolysis, and phenylpropanoid biosynthesis were enriched and the candidate genes targeting to the disease resistance in the plant were also inferred.

## Results

### RNA sequencing and *de novo* assembly

To understand the molecular basis of the disease resistance of *O. meyeriana* against *Xoo*, total RNAs from the leaves at 0 h, 1 h, 2 h, 4 h, 24 h, and 48 h after *Xoo* inoculation were isolated and used for high-throughput sequencing. The RNAs were pooled into three groups: CO acts as the control; TE represents the RNAs from 1 h, 2 h and 4 h after inoculation as the early response; TO signifies the late response derived from the 24 h and 48 h RNA samples. Each group was further divided 3 sub-samples. Altogether, 9 sub-samples were applied to Illumina HiSeq2000 platform for sequencing. The sequencing data are summarized (Table 1) and total 58.7 Gb 100-base clean reads were obtained.

The short reads pooled from the three libraries were primarily assembled into 210,601 contigs using the Trinity package. Prediction of ORFs was carried out to obtain 97,596 contigs encoding ORFs with 4 types (no-start-codon, no-stop-codon, no-start-stop-codon and intact ORFs). All the contigs encoding ORFs were further assembled using the software CAP3, resulting in 57,313 unitranscripts with a mean length of 2,637 bp (Table 1). All the 57,313 unitranscripts were annotated by sequence similarity using BLASTx against the NCBI non-redundant (NR) protein database. In a total of 55,671 (97.14%) unitranscripts have at least one significant alignment to existing gene models in the NR database at an E-value cut-off of 1e-5. The distribution and abundance of the transcripts in species had the top three matches of *O. sativa, O. brachyantha*, and *Brachypodium distachyon.*

### Identification of differentially expressed unitranscripts (DEUs)

**Figure 1:**
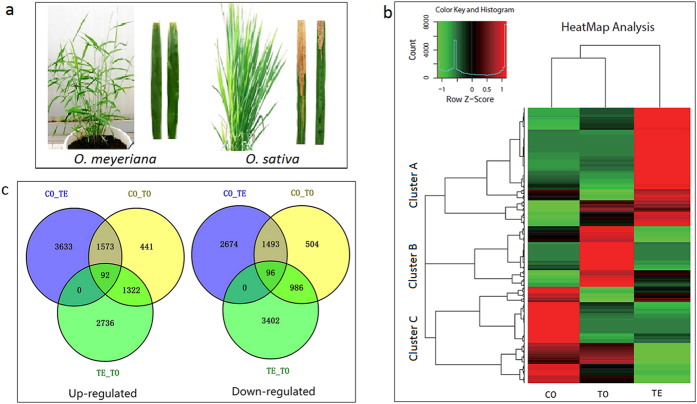
Comparison of leaf symptoms of *Oryza meyeriana* and *O. sativa* inoculated with *Xanthomonas oryzae* pv*. oryzae* and (**a**) the expression patterns of the differentially expressed unitranscripts (DEUs) of *O. meyeriana* in response to *X. oryzae* pv*. oryzae (Xoo*) attack. (**b**) Heat-map depicting the expression changes in all the DEUs. (**c**) Venn diagram exhibiting the DEUs’ distribution in three samples.

The relative expression level of each transcript in terms of fragment per kilobase per million (PKFM) was estimated by mapping all the clean reads from each library back to the 57,313 transcripts. Consequently, 14,143 DEUs (24.7%) were identified between the three libraries of CO, TE and TO with *P* values ≤0.05 (Table S1). The analysis of DEU profiles across the three libraries indicated that the DEUs were roughly clustered into three main groups (Fig. 1b). Cluster A comprised the transcripts mostly up-regulated at an early stage; cluster B contained the transcripts mostly up-regulated at a late stage, and cluster C mostly down-regulated after *Xoo* infection.

To find out the intrinsic differences in the transcriptome of the three libraries, DEUs in the libraries of TE and TO were compared with the control (CO) (Fig. 1c). Totally, 9,561 (5,298 up-regulated and 4,263 down-regulated) and 6,507 (3,428 up-regulated and 3,079 down-regulated) DEUs were identified between TE/CO and TO/CO, respectively. Meanwhile, 8,634 (4,150 up-regulated and 4,484 down-regulated) DEUs were also found between TE/TO. However, 92 DEGs were common between TE/CO and TE/TO. It is noteworthy that 3,633 DEUs were discovered up-regulated solely in TE compared with CO.

Furthermore, the functional classification of the DEUs in *O. meyeriana* after *Xoo* infection was analyzed using GO (Gene Ontology). A total of 8,134 (57.6%) transcripts were assigned to at least one GO term. Among the other biological processes, where the term occurrences took place, metabolic process (25.6%) and cellular process (23.4%) were highly represented (Fig. S1). In addition, transcripts participation or involvement in biological process like response to stimulus, immune system, cell death, were recognized through GO annotation.

To explore the biochemical pathways in which DEUs were involved, the metabolic pathway analysis was performed for all the DEUs using the KEGG Automatic Annotation Server (KAAS). The results revealed that 5,137 DEUs was assigned into 313 Kyoto Encyclopedia of Genes and Genomes (KEGG) pathways (Table S2). Most of the DEUs were involved in the primary metabolisms, notably biosynthesis of amino acids, purine metabolism, and carbon metabolism, etc. Most importantly, several pathways associated with disease defence were also enriched by KEGG which would be stressed on in more detail below.

### Differentially expressed genes (DEGs) involved in the plant-pathogen interaction pathway

**Figure 2:**
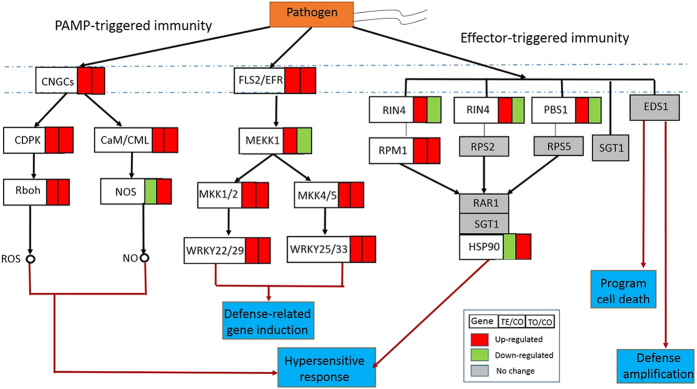
Key enzymes encoded by the differentially expressed genes (DEGs) involved in the plant-pathogen interaction pathway enriched by KEGG analysis.

In the plant-pathogen interaction pathway (Ko04626), 19 DEGs containing 133 DEUs were identified as up-regulated at the early stage in response to *Xoo* inoculation (Fig. 2 and Table S3). Except WRKY1 (WRKY transcriptional factor 1), PTI1 (pto-interacting protein 1) and CREK1 (chitin elicitor receptor kinase 1), 16 DEGs were enriched in two immune signaling pathways: the PTI and ETI. However, 6 DEGs involved in PTI were up-regulated at the early stage (Fig. 2). In plants, FLS2 (flagellin-sensing 2) perceives the bacterial flagellin, and then activates a signaling tandem of the mitogen-activated protein kinase (MAPK) cascades, like MEKK1 (mitogen-activated protein kinase kinase kinase 1), MKK1/2 (mitogen-activated protein kinase kinase 1), and MKK4/5 (mitogen-activated protein kinase kinase 4/5). Finally, the transcriptional factors of WRKY22/29 (WRKY transcription factor 22) and WRKY25/33 (WRKY transcription factor 25) were activated, which in turn induce expression of the defence-related genes^18^,^19^.

PAMP usually triggers a rapid increase in the cytoplasmic Ca^2+^ concentration and activates the calcium-dependent protein kinase (CDPK) in plant cells^26^. In the present study, in *O. meyeriana*, 4 genes involved in Ca^2+^ signaling were up-regulated at an early stage of *Xoo* infection (Fig. 2 and Table S3). These DEGs are the cyclic nucleotide gated channels (CNGCs), CDPK, calmodulin (CaM), and calmodulin-like proteins (CML). Additionally, 11 transcripts encoding nitric oxide synthase (NOS) were enriched but only the minority showed up-regulated expression in the early and late stage after *Xoo* inoculated (Table S3). The calcium signaling has long been demonstrated to be related to response to the pathogen attack, which is usually accompanied by an increase of both ROS and NO^26^.

The secondary immune response known as ETI is thought to mount a second layer of defence^18^,^19^. The second signaling pathway is composed of five genes encoding the receptor proteins to perceive the bacterial effector proteins. Here, three DEGs encoding receptor proteins (RPM1, the disease resistance protein RPM1; RIN4, PRM1-interacting protein 4; PBS1, the disease resistance protein PBS1) showed up-reregulated gene expression pattern at the early stage of *Xoo* infection (Fig. 2). In addition, the downstream HSP90 (heat shock protein 90) was found down-regulated at the early stage (TE/CO) followed by up-regulated at the late stage (TO/CO). HSP90 has been reported to form a complex with RAR1 (disease resistance protein RAR1) and SGT1 (suppressor of G2 allele of SKP1), which may interact with the R proteins^27^, perhaps to make a contribution to the observed HR in *O. meyeriana*^12^,^13^.

### DEGs in the phytohormone and ubiquitin-mediated pathways

Previous studies have demonstrated that various disease resistance gene are involved in phytohormone signal transduction and ubiquitin-mediated proteolysis^28^,^29^. With a view to indentifying genes involved in phytohormone signal transduction and ubiquitin-mediated proteolysis, gene expression was calculated. As expected, 44 DEGs encoding the proteins involved in these signaling pathways were detected (Table S4).

**Figure 3:**
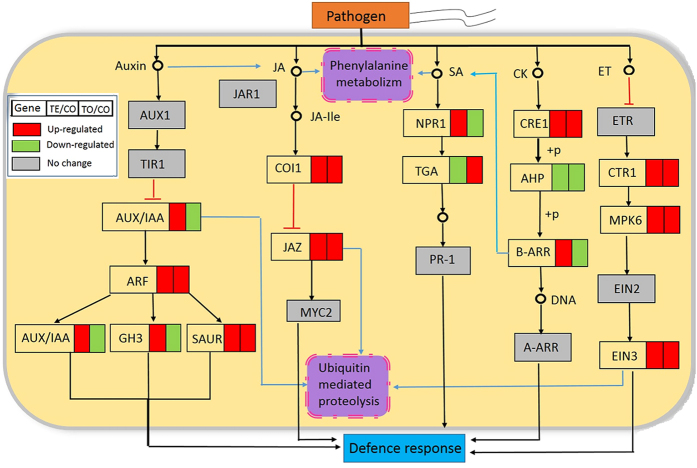
Differentially expressed genes (DEGs) involved in the phytohormone signaling pathways in response to *Xoo* infection.

Phytohormones, such as salicylic acid (SA), jasmonic acid (JA), gibberellic acids (GAs), ethylene (ET), and brassinosteroids (BRs), act as signaling molecules to trigger or mediate a diverse array of plant immune responses^28^. In this study, 8 DEGs related to SA, JA, and ET signaling pathways were enriched in *O. meyeriana* in response to *Xoo* infection (Fig. 3). In JA signaling, two DEGs (encoding the coronatine insensitive protein 1, COI1; jasmonate ZIM-domain containing protein, JAZ) were enriched to be up-regulated. The other genes encoding JAR1 (jasmonic acid-amino synthetase) and MYC2 (transcription factor MYC2) involved in JA signaling did not respond to *Xoo* infection. It is well-known that SA is also an important phytohormone that regulates plant defence processes. NPR1 (regulatory protein NPR1) is an essential regulator in SA-signaling was up-regulated at the early stage in *O. meyeriana*. The downstream gene encoding the transcription factor TGA was showed significantly different expression at different stages, which was down-regulated between TE/CO, while up-regulated between TO/CO (Fig. 3 and Table S4).

As one of the backbone of the induced defence signaling pathway, ET signal transduction has been well documented^30^. In our study, the genes encoded with serine/threonine-protein kinase CTR1, mitogen-activated protein kinase 6 (MAPK6), and ethylene-insensitive protein 3 (EIN3) were shown up-regulated between TE/CO. In contrast, *Xoo* infection did not induce the expression of genes encoding ethylene receptor ETR and ethylene-insensitive protein 2 (EIN2).

Recent studies indicated that the auxin and cytokinin (CK) signaling pathways may be involved in defence response^31^. Here, several DEGs involved in auxin and CK signaling pathways were also identified (Fig. 3).

Ubiquitination exists extensively in plants and may be involved in the regulation of plant disease resistance^32^. In this study, 4 DEGs encoding E2 were identified between TE/CO after *Xoo* infection, among them, three showed up-regulated and another one showed down-regulated (Table 2). Eighteen DEGs encoding E3 ubiquitin ligase belonging to five types were identified (Table 2), 9 of which were up-regulated between TE/CO. Especially, 7 DEGs out of the 18 belong to the type of the multi subunit RING-finger, which were shown to mediate plant disease resistance^33^.

### DEGs in the phenylpropanoid biosynthesis pathway

**Figure 4:**
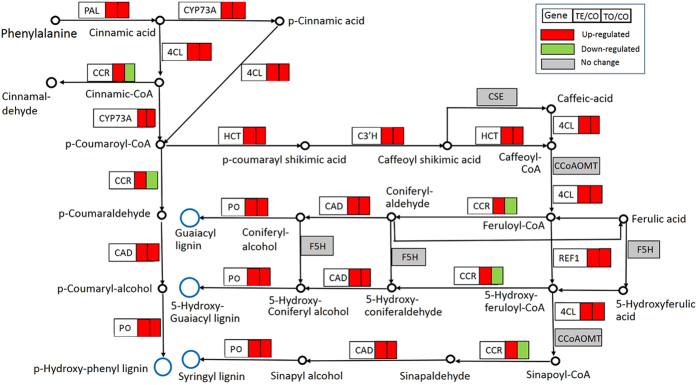
Differentially expressed genes (DEGs) involved in the phenylpropanoid metabolic pathway in response to *Xoo* infection.

The phenylpropanoid biosynthesis pathway is one of the important secondary metabolic pathways in the plant, which plays important roles in plant defence^34^. Phenylpropanoid biosynthesis pathway of *O. meyeriana* in response to *Xoo* infection was annotated by KEGG analysis (Fig. 4). At most 128 unitranscripts encoding 12 DEGs were shown to be up-regulated especially at the early stage after *Xoo* infection (Table S5). In phenylpropanoid pathway, the first key enzyme is the phenylalanine ammonia-lyase (PAL), which was well known to be induced in many plants by pathogen attack. Here, all the 14 transcripts encoding PAL were up-regulated between the TE/CO. Further, there are a large number of enzymes involved in lignin biosynthesis^34^. Although it is not clear which transcript encodes this specific enzyme, the majority of the transcripts encoding 4-coumarate-CoA ligase (4CL), cinnamoyl-CoA reductase (CCR), cinnamyl-alcohol dehydrogenase (CAD), and peroxidase (PO) were shown to be up-regulated after *Xoo* infection (Fig. 4 and Table S5).

### Enriched *R* genes in response to *Xoo* infection

The *R* genes, which encode disease resistance proteins especially containing the LRR/NBS-LRR domains, are widely recognized to defeat pathogens in many plants^35^. In rice, most of the *R* genes especial to BB are unique and recessively regulated^36^. Previously, more than 40 resistance genes (*Xa*) have been identified, conferring resistance to *Xoo* in rice^7^. In *O. meyeriana*, two *Xa* genes (*xa13* and *Xa21*) were identified as DEGs after *Xoo* infection (Table S6). There is only one transcript annotated as the disease resistant allele *xa13*, which exhibited high expression at the early stage and continue to rise at the late stage after *Xoo* infection.

*Xa21*, which encodes a receptor-like kinase, is a broad-spectrum resistance gene of rice against BB^37^. Here, 14 and 3 transcripts were identified and annotated to encode *Xa21* and *Xa21*-binding proteins, respectively (Table S6). Among the 17 transcripts encoding *Xa21*-related proteins, 8 were up-regulated between TE/CO, while 6 were continuously up-regulated between TO/TE. It is suggested that most of the *Xa21* transcripts were stimulated by the *Xoo* infection.

The *R* genes occur ubiquitously in the plant kingdom and comprise the second mechanism of disease resistance by recognizing the pathogen *Avr* gene directly or indirectly^38^. Until now, five different types of *R* genes have been identified up to date^39^. A large number of *R* genes were identified as the family that encoded NBS-LRR containing domain protein^38^. In this study, 103 transcripts encoding various *R* genes were enriched in *O. meyeriana* after *Xoo* infection (Table S6). Among them, 42 DEUs belonging to the NBS-encoding *R* genes, were detected in the transcriptomic profile (Table S6). Some of the NBS-encoding *R* genes were also detected to encode other domains, such as TIR and CC domain attached to the *N*-terminal. However, different expression patterns were observed for these transcripts encoding NBS-containing domain, some of them were down-regulated and the rests were up-regulated in response to the *Xoo* infection (Fig. S6).

In addition, many DEUs associated with the disease resistance genes annotated to the NBS-encoding transcripts. At least, five groups could be detected in terms of the annotation information (Table S6). The first group is consisted of the 6 transcripts encoding disease resistance gene analog (RGA) proteins^40^, of which two transcripts were up-regulated and 3 down-regulated at the early stage. The second group (24 transcripts) was annotated to encode the rust-related R proteins, such as resistance kinase and stripe rust resistance protein. Of these, 17 transcript were up-regulated and 5 showed lower expression in TE or TO. The third group is those to encode nb-arc domain. Only one transcript was up-regulated in TE compared with CO while the other three transcripts were down-regulated between TE/CO. The fourth group was annotated to encoding negative regulator of systemic acquired resistance, all of the 3 transcripts showed down-regulated at the early stage. The final group was consisted of 24 transcripts encoding proteins relative to various resistance, of which some showed up-regulated and the others down-regulated.

### Validation of the selected DEUs by RT-qPCR

**Figure 5:**
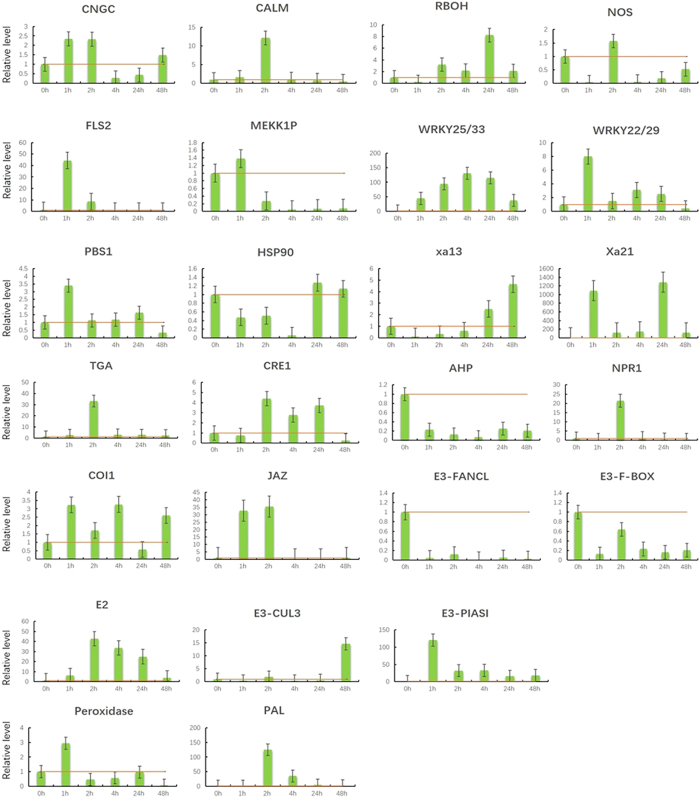
Validations of the differentially expressed transcripts by RT-qPCR. Samples were collected from the leave at 0, 1, 2, 4, 24, and 48 h after *Xoo* infection. All data were normalized to the expression level of β-actin. The data represent the change fold at each time point in the infected samples vs. the control sample (Time 0). Bars show the standard deviations from three replicates.

Twenty-five DEUs involved in the plant innate immunity, phytohormone signaling, phenylpropanoid biosynthesis pathway, and the *R* genes were selected for RT-qPCR using the specific primers to confirm the reliability and availability of DEUs obtained from the RNA sequencing (Table S7). The relative expression levels of the selected genes were determined at 0, 1, 2, 4, 24, and 48 h after *Xoo* inoculation. The RT-PCR results (Fig. 5) showed that 9 genes involved in the plant innate immunity, were up-regulated at an early stage (1–2 h), and thereafter showed decreased expression at the later stage (24–24 h) of *Xoo* infection. Only the transcript encoding RBOH continued to rise until 24 h post-inoculation and the *HSP90* was down regulated at the early stage of infection. These results further indicated that the innate immune response arose at early stage of *Xoo* infection in *O. meyeriana*. The similar pattern was observed for these transcripts involved in JA and SA-mediated signaling pathways. Various E3 genes showed different expressing responses.

## Discussion

In fact, immune machinery may widely exist in the plant world. More importantly, the expression of the resistance is mostly dependent upon how the common signaling machinery is used in a given plant-pathogen interaction^41^. Therefore, RNA-seq was used to get clear understanding of how the wild rice *O. meyeriana* responded to the *Xoo* infection. In this study, 327 Mb of transcripts in length was obtained by assembling 58.7 Gb clean reads. Finally, 57,313 unitranscripts encoding ORF were *de novo* assembled.

By bioinformatic analysis of the transcriptomic data, we provide novel insights into the defence responses of *O. meyeriana* to *Xoo* infection. Moreover, several metabolic pathways related to disease resistance were significantly enriched. In plants, the innate immunity is triggered through PTI, and then ETI, which provides the first line of disease resistance^18^,^19^. In the case of *O. meyeriana*, the expression level of all these genes involved in PTI was up-regulated at the early stage after *Xoo* inoculation (Fig. 2 and Table S3). These DEGs may comprise an FLS2-mediated plant immune pathway to confer *O. meyeriana* against *Xoo* infection. In addition, we have manually detected 4 transcripts encoding the brassinosteroid insensitive 1-associated receptor kinase (BAK1) being up-regulated after *Xoo* infection (Table S3). BAK1 was previously reported to form a flg22-induced complex with FLS2, which was required for all known downstream flg22-signaling responses in *Arabidopsis thaliana*^42^. However, two members of MPK3/6 and MPK4 involved in the MAPK cascades were not identified in this wild rice. In fact, MAPK cascades have been reported to play important roles, especially in dicot plant responses to pathogen infection. Further, a large number of MAPK genes were also demonstrated to be differentially expressed in response to *Xoo* infection in both of the resistant and susceptible rice lines^43^. In terms of these missed components of MAPK cascades, the innate immunity of *O. meyeriana* may be different from that of *A. thaliana* and rice.

In compatible plant-pathogen interaction, the bacterial pathogen may secret effectors into the plant cells to suppress the PTI^44^. However, plants adopt the second pathogen-sensing mechanism called ETI to defeat the effectors. During ETI, the effector proteins are recognized by a class of plant receptor proteins that usually contain NB-LRR domains^18^,^19^. Up to now, several receptor proteins such as RIN4, RPM1, EDS1, have been identified in various plants^19^. In *O. meyeriana*, at least three genes encoding RIN4, RPM1, PBS1 were enriched as up-regulated at the early stage in response to *Xoo* infection (Figs 2 and 5). Conclusively, our data suggest that the innate immunity is an early responding event and may comprise a basal resistant mechanism to defeat *Xoo* attack in the incompatible *O. meyeriana-Xoo* interaction.

In addition, the receptor-kinase of flg22 also triggers Ca^2+^ signaling by FLS2^26^. Almost the whole components (CNGC, CDPK, CaM/CML, Rboh, and NOS at least for partial transcripts) involved in the Ca^2+^ signaling were shown to be up-regulated in *O. meyeriana* in response to *Xoo* infection (Fig. 2 and Table S4). Previous studies showed that the crucial role for the induction of Ca^2+^ signaling in response to abiotic and biotic stresses^26^. In rice, Ca^2+^ signaling was also identified as up-regulated in response to the infection of several pathogens by the microarray-based transcriptomic studies^45^. One of the outcome of Ca^2+^ signaling is HR, which is one of the hallmarks of the plant innate immunity, especially in the incompatible systems^46^. HR was observed on the *Xoo*-inoculated leaves of *O. meyeriana*, which suggested the presence of immunity in *O. meyeriana*^11^. HR is usually associated with persistent production of ROS, NO, SA and Ca^2+^ fluxes^26^. The increase of NO especially in the xylem cell walls in *O. meyeriana* was indeed observed after *Xoo* inoculation by the immunohistochemistry assay^13^. Therefore, we believe that the observed increase of HR and NO in the *O. meyeriana* leaves inoculated with *Xoo* can be ascribed to the inducible expression of these genes involved the innate immune and Ca^2+^ signaling pathways.

Phytohormones, such as JA, SA, ET, etc., play key roles in the complex signaling cascades in growth, development, as well as defence responses. Several DEGs involving in these phytohormone signaling pathways were enriched (Fig. 2), suggesting that JA, SA and ET signaling pathways may be included in the response of *O. meyeriana* to *Xoo*. SA is usually effective against biotrophic pathogens; whereas JA and ET mainly involve in defence of necrotrophic pathogens. And SA-JA antagonism was observed in *O. sativa* and *A. thaliana*^47^. For example, overexpression of *OsNPR1* led to activation of SA-response genes and the concomitant suppression of JA maker genes in rice^48^. However, both SA and JA pathways were up-regulated in *O. meyeriana* after *Xoo* infection. Similar results were also reported in a resistant rice line carrying *Xa39*, but not in its susceptible parental lines^23^. However, only JA-signaling genes were up-regulated after inoculation of pathogen in soybean-*X. axonopodis* pv. *glycines* pathosystem^24^. Even no genes involved in both SA or JA-signaling was enriched in peach leaves inoculated with the invasive *X. arboricola* pv. *pruni*^25^. Therefore, response of SA- and JA-signaling to microbial pathogens seems different and may be dependent upon various pathosystems.

The ubiquitin-proteasome system (UPS) play important roles in various cellular processes, which contains four basal components of E1, E2, E3 and ubiquitin^49^. Plant genomes usually encode large numbers of E3 ubiquitin ligase. In fact, UPS-mediated protein degradation has been demonstrated in regulation of almost all of hormone signaling pathways, such as the auxin, CK, ET, JA and SA^50^. For example, the E3 ubiquitin-ligase SCF^COI1^ complex may activate with the downstream response of the JA signaling through degradation of the JAZ repressor^51^. Furthermore, many E3 ligases were also identified to participate in the regulation of plant immunity^52^. In our study, up to 19 unitranscripts encoding various types of E3 ubiquitin ligase were revealed to be differentially expressed in response to *Xoo* infection (Table 2). We believe these E3 ubiquitin ligases may play an important role in the defence response after *Xoo* infection in *O. meyeriana*, perhaps through cross-talking with the other disease resistance pathways, like phytohormone and plant immune signaling pathways.

The secondary phenylpropanoid metabolic pathway was revealed to be overall up-regulated. The similar results have been reported in many plant-pathogen interactions^53^. This pathway can synthesize many small molecules and strengthen the cell wall by depositing callose and lignin, which may inhibit the pathogenic bacteria growth and spread in plants^34^. In *O. meyeriana*, thickening of cell wall especially in the leaf xylem tissue was reported as a defence mechanism against *Xoo*^12^. Therefore, induction of phenylpropanoid pathway by *Xoo* infection may contribute to the cellular wall thickening of the leaf xylem tissue in *O. meyeriana*.

The *R* genes are regarded as the second mechanism of disease resistance and have been recognized in various plants. To date, about 40 *R* genes specific to BB have been identified in rice, and nine *(Xa1, Xa3/Xa26, xa5, xa13, Xa10, Xa21, Xa23, xa25* and *Xa27*) have been isolated by map-based cloning^7^. Two known genes of *Xa21* and *xa13* were identified as DEGs in this work, but not in previous studies^17^. In addition, our data revealed more than 100 unitranscripts encoding various *R* genes that were differentially expressed in response to *Xoo* infection (Table S6). Among them, the majority of these DEUs encode the typical R proteins containing NBR domain.

In conclusion, the transcriptomic analysis provide us with a glimpse of the transcriptional response of *O. meyeriana* to *Xoo* attack, indicating that several resistant-related signaling pathways were up-regulated and consisted of a complex defence network. The plant innate immune (MTI and ETI) may comprise the basal defence frontier, and then activates the *R* gene expression, which in turn induces HR and thickening of xylem cell wall to restrict spreading of the bacterial cells in the leaf xylem tissue of *O. meyeriana*.

## Methods

### Plant materials

*O. meyeriana* was collected from the native niches at Yuanjiang county, Yunnan province of China, and grown in a greenhouse. For one individual plant, three leaves were inoculated with *X. oryzae* pv*. oryzae* at an inoculum concentration of 3 × 10^8^ cells per milliliter by the leaf clipping method^54^. After inoculation, the leaves were collected separately at, 1 h, 2 h, 4 h, 24 h and 48 h, immediately frozen in liquid nitrogen, and then stored at −80 °C for isolation of the total RNAs. The leaves sampled immediately after inoculation with the same *Xoo* cell suspension, were served as the control (0 h). Three replicates of leaf samples (3 × 3 leaves) were collected for each time points.

### RNA extraction and library construction

Total RNAs were extracted from all the leaf samples using Trizol reagent (Roche, Switzerland) according to manufacturer’s instructions. The genomic DNAs were removed by DNase I (Fermentas, USA). The RNA concentration and quality were measured with the Qubit fluorometer (Invitrogen, USA) and the Agilent 2100 Bioanalyzer, respectively. Only the RNA samples with a RIN (RNA integrity number) >8.0, OD_260/280_ ratio ranged from 1.9 to 2.1, and OD_260/230_ ratio of 2.0 to 2.5, were used for the next processing.

For RNA sequencing, the RNA samples were combined into three groups: CO, TE and TO, which were designed for the control leaves (0 h); early stage (1 h, 2 h and 4 h); and the late stage (24 h and 48 h), respectively. Each group was equally divided into 3 sub-samples. For each sub-sample, the mRNAs were further purified from the total RNAs (20 μg) using Dynaloligo (dT) 25 magnetic beads and fragmented into smaller pieces. The fragmented mRNAs were used for the first-strand cDNA synthesis with the random primer by reverse transcriptase. Second strand cDNAs were synthesized using RNase H and DNA polymerase I. The resulting cDNA fragments were further repaired at 3′-end by adding a single base (A), and then ligated with the Illumina adapters. Subsequently, the cDNA fragments were purified and enriched by PCR. The quality of cDNA libraries was measured by Agilent 2100 Bioanalyzer.

### Illumina sequencing and *De novo* assembly

The cDNA libraries were sequenced using the Illumina HiSeq2000 platform. The fluorescent image processing, base-calling and calculation of quality value were performed by the Illumina data processing pipeline 1.4. Meanwhile, sequencing quality was assessed by fastQC (http://ww.bioinformatics.bbsrc.ac.uk/projects/fastqc/). Finally, 100 bp paired-end (PE) clean reads were obtained.

The sequence assembly was performed on a server with 24 cores and 256 GB random access memory. All the PE reads derived from the above 9 libraries were *de novo* assembled using the Trinity_release_20131110^55^ under default parameters. In order to realize better results in the following analysis, the best candidate coding sequence (CDS) analysis was performed for each contig using the perl script in the Trinity package. All the contigs with potential CDS were further reassembled using software CAP3^56^ to generate a non-redundant set of unitranscripts. The parameters used for the reassembly were >95% identical with a minimum of 40 bases with maximum of 20 bases of unmatched overhangs at the sequence end.

To assay the assembly quality, all the PE reads were aligned back to these contigs by using Bowtie2 program (v2.0.0-beta5)^57^, and the aligned rate was calculated. The common perl scripts were used to assess the length distribution of the transcripts, N50 number, average length, max length, and the numbers of contigs in different length intervals.

### Functional annotation

All the reassembled contigs with predicted ORFs generated by CAP3 were used for similarity search against the NR database downloaded from GenBank (http://www.ncbi.nlm.nih.gov/) by using local blastx program with the expect E-value cut-off <10^−5^.

The blast results were imported into the Blast2GO^58^ and performed functional annotation. Gene ontology (GO) enrich was achieved on WEGO (http://wego.genomics.org.cn/cgi-bin/wego/index.pl). Meanwhile, Kyoto Encyclopedia of Genes and Genomes (KEGG) pathways were assigned to each contig on KEGG Automatic Annotation Server (KAAS) (http://www.genome.jp/kegg/). Thus, multiple contigs could be clustered to the same GO terms and the same KEGG pathway.

### Abundance estimation and identification of differential expressed genes

In order to calculate the relative express level of each contig in different samples, all the PE reads pooled from the three sub-samples in each sample group were aligned back to the final annotated contigs by using perl scripts in the Trinity package under default parameters option^55^. The digital expression levels from the alignment for each contig were normalized using RESM-based algorithm by using perl scripts in Trinity package. So values of the fragments per kilobase per million (FPKM) for each contig were obtained^59^. And then, the edgeR package (the Empirical analysis of Digital Gene Expression in R)^60^ was used to extract those contigs which were regarded as differentially expressed genes with the *P*-value ≤ 0.05 and the log_2_fold-change (log_2_FC) >1.

### Validation of the selected genes by qPCR

Twenty five DEUs were selected to verify the reliability of the RNA sequencing data by quantitative real-time PCR (RT-qPCR). The designed primers for amplification of the selected transcripts were presented in Table S7. Total RNAs were isolated from the *O. meyeriana* leaves at 0 h, 1 h, 2 h, 4 h, 24 h and 48 h post inoculation with *Xoo* as described above. After treated with DNase I, first strand cDNAs were synthesized using oligo (dT) and random hexamers as primers by M-MLV reverse transcriptase (Tiangen Co., Beijing, China) according to the manufacturer’s instructions. Real-time PCR were performed in a 25 μl volume using SYBR premix Ex Taq^TM^ II (Takara Co., Dalian, China) on the CFX Connect Real-time PCR detection system (Bio-Rad, Hercules, USA). The quantitative PCR was followed by the program: 5 min at 95 °C; followed by 45 cycles of amplification with denaturation for 5 *s* at 95 °C, annealing for 30 *s* at 55 °C, and extension for 20 *s* at 72 °C. Triplicate under identical conditions were synchronously performed for all selected genes. The relative expression values of each transcript were then calculated by the delta-delta Ct (2^−ΔΔCt^) method using the CFX Manager 3.0 of the amplifier.

### Data availability

Raw sequencing data are available through the NCBI Sequence Read Archive (BioSample Submission Potal, accession number: SRP071037). All samples were sequenced as 100 bp paired-end reads on an Illumina HiSeq2000 sequencer.

## Additional Information

**How to cite this article**: Cheng, X.-J. *et al*. Transcriptome analysis confers a complex disease resistance network in wild rice *Oryza meyeriana* against *Xanthomonas oryzae* pv. *oryzae. Sci. Rep.* **6**, 38215; doi: 10.1038/srep38215 (2016).

**Publisher’s note:** Springer Nature remains neutral with regard to jurisdictional claims in published maps and institutional affiliations.

## Supplementary Material

Supplementary Figures

Supplementary Dataset 1

## Figures and Tables

**Table 1:** Summary of the sequencing data and *de novo* sequence assembly of *Oryza meyeriana* transcriptome in response to *Xanthomonas oryzae* pv. *oryzae* infection.

Sample	Raw data		Clean data			
	Reads	Gb	Reads	Gb	Assembly statistics	
CO1	76,825,162	7.68	71,699,288	7.17	Total no. of contigs	210,601
CO2	69,922,902	6.99	64,185,172	6.42	No. of contigs with ORF	97,596
CO3	77,747,124	7.77	70,430,752	7.04	No. of unitranscritpts with ORF	57,313
TE1	79,450,850	7.94	72,647,958	7.26	Total size (Mb)	151.1
TE2	66,303,800	6.63	59,980,114	5.99	Maximum length (bp)	18,852
TE3	76,413,118	7.64	69,886,360	6.99	Average length (bp)	2,637
TO1	59,733,550	5.97	53,460,904	5.34	N50 length (bp)	3,123
TO2	70,975,938	7.09	63,882,078	6.39	Contigs with hits in NR	55,671
TO3	66,307,936	6.63	61,474,994	6.15	Differentially expressed unitranscripts	14,143
Total	643,680,380	64.4	587,647,620	58.7		

**Table 2:** Candidate ubiquitin ligases encoded by the differentially expressed unitranscripts (DEUs) of *Oryza meyeriana* in response to *Xanthomonas oryzae* pv. *oryzae* infection at the early stage.

Enzyme	Type	Protein	Ko ID	Number of unitranscripts		
				Total	Up-regulated	Down-regulated
E2		UBE2D	K06689	1	1	0
		UBE2G1	K10575	1	1	0
		UBE2H	K10576	1	0	1
		UBE2O	K10581	2	1	1
E3	HECT	TPIP12	K10590	2	2	0
	Single RING- finger	SIAH1	K04506	1	1	0
		RCHY1	K10144	3	3	0
		PIAS1	K04706	1	1	0
		FANCL	K10606	7	1	6
	Multi subunit RING- finger	CUL1	K03347	1	1	0
		SKP1	K03094	1	1	0
		FBXW1_11	K03362	4	4	0
		SKP2	K03875	2	1	1
		CUL3	K03869	4	2	2
		DDB2	K10140	1	1	0
		SKP1	K03094	2	2	0
	APC/C	APC1	K03348	2	1	1
		APC4	K03351	5	3	2
		APC8	K03355	2	1	1
		APC3	K03350	4	3	1
		APC5	K03352	5	2	3
		APC7	K03354	2	0	0
