# Supplementary material for: Transcriptome analysis confers a complex disease resistance network in wild rice Oryza meyeriana against Xanthomonas oryzae pv. oryzae
Source: Sci Rep. 2016 Dec 1;6:38215. doi: 10.1038/srep38215 (PMC5131272; doi:10.1038/srep38215)
Supplement: Supplementary Figures [file srep38215-s1.pdf]

Transcriptome analysis confers a complex disease resistance network in wild rice *Oryza meyeriana* against *Xanthomonas oryzae* pv. *oryzae*

Xiao-Jie Cheng[1], Bin He[1], Lin Chen[2], Su-Qin Xiao[2], Jian Fu[2], Yue Chen[2], Teng-Qiong Yu[2], Zai-Quan Cheng[2] & Hong Feng[1]

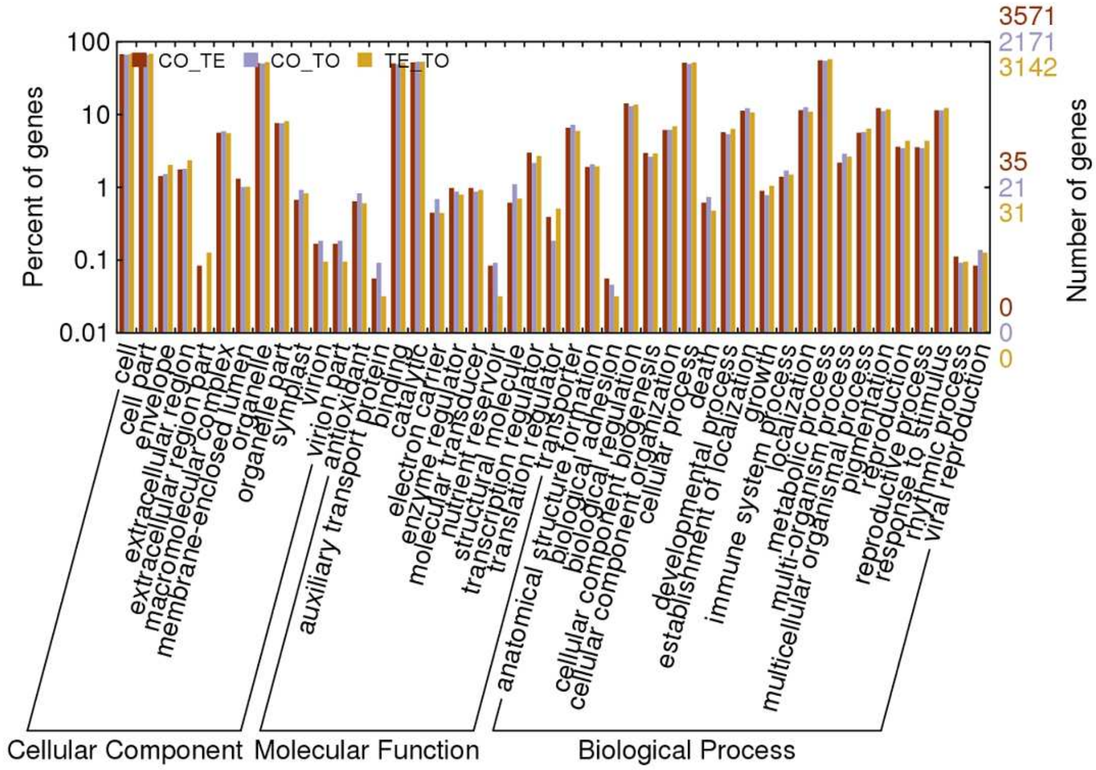

Figure S1. **GO functional categorization of differentially expressed unitranscripts (DEUs) in three samples after *Xoo* inoculation.** Numbers of DEUs assigned to the same GO term are indicated by the bars; the x-axis indicates the subcategories, and the y-axis indicates the number of genes in a category. TE and TO represent the *O. meyeriana* leaf sample at the early stage (1-4 h) and the late stage (12-24 h) post *Xoo* inoculation. CO shows the leaf sample without pathogen inoculation.

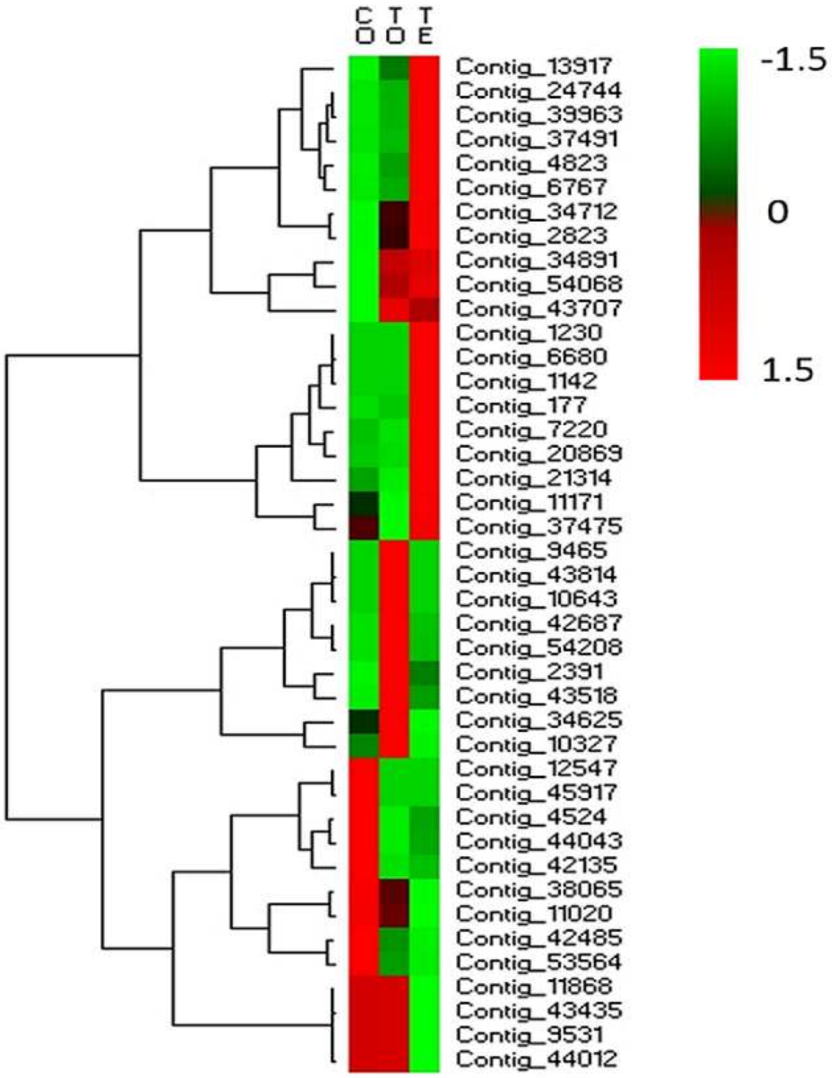

Figure S2. Hierarchical cluster analysis of 42 differentially expressed unitranscripts encoding R proteins containing the nucleotide-binding site/leucine-rich repeat (NBS-LRR) domain.
